# The Heart Team Expands

**DOI:** 10.1016/j.shj.2022.100121

**Published:** 2022-11-21

**Authors:** Anthony DeMaria

**Affiliations:** Judy and Jack White Chair in Cardiology, Sulpizio Cardiovascular Center, University of California, San Diego, La Jolla, California, USA

Looking back, the historical evolution of the heart team is somewhat unclear. The referral of patients from internists (cardiologists) to surgeons (cardiac surgeons) has always existed. Inherent in this interaction was the assumption that both had considered and agreed about the best management for a patient. However, this collaboration became much more interactive and important as cardiologists began applying percutaneous interventions to treat conditions for which surgery had been the only alternative. Coronary artery disease was, of course, the major stimulus for direct communication and interaction between physicians in selecting treatment and primarily involved interventional cardiologists and surgeons. The concept of a heart team, that is, a collaboration of a group of physicians and health care professionals involved in selecting the optimal care of a patient, was first formalized in 2010 by the European Society of Cardiology specifically for myocardial revascularization.^1^ However, the heart team became even more important and relevant with the advent of percutaneous catheter procedures for structural heart disorders. Cardiac imagers, radiologists, anesthesiologists, and others became more intimate and contributing members of the heart team. Recently, the development of novel interventional procedures to treat heart failure has led to an expansion of the heart team to include specialists in advanced heart failure.

A number of procedures have been applied for the electrophysiological abnormalities associated with heart failure. Cardiac resynchronization therapy has proven beneficial to patients with abnormal conduction associated with failure. In addition, intracardiac defibrillation devices have been effectively employed for ventricular arrhythmias. These procedures have resulted in a collaboration between electrophysiologists and heart failure specialists but usually in a referral for a procedure that was deemed indicated and directed primarily to the electrical abnormality. Such patients have not usually come to the attention of the heart team.

A variety of interventional procedures for heart failure due to a structural disease have existed for some time. Cardiac surgery has been the traditional therapy for patients with heart failure due to a disease of any of the heart valves since the 1970s. Transcatheter aortic valve replacement for aortic stenosis was the first transcatheter therapy for heart failure due to valvular heart disease. Similarly, coronary bypass surgery and coronary angioplasty and stenting have been applied for heart failure due to a coronary disease. However, in both cases, surgical and percutaneous approaches were directed toward the pathology of the valves and/or coronaries causing the heart failure. Although surgical valve replacement and repair has been applied for functional mitral regurgitation due to left ventricular dysfunction, the benefit has been equivocal. Surgical edge-to-edge mitral plication for regurgitation has been applied in the past with some success by Alfieri et al.^2^ and subsequently accomplished by transcatheter techniques (transcatheter edge-to-edge repair). In the process, edge-to-edge repair represented the first interventional approach for valve abnormalities which were not the primary mechanism (i.e., myocardial dysfunction) of the failure.

A variety of devices have been and continue to be developed that are suitable for surgical or transcatheter implantation for different grades of heart failure. For patients with advanced heart failure or shock, several mechanical cardiac support devices exist or are being investigated. A spectrum of techniques from simple aortic balloon counter pulsation to external membrane oxygenation are being used to support patients with severe levels of failure. More such devices are being developed for these critically ill patients. These technologies lend themselves to percutaneous application and, therefore, often involve the heart team.

A greater number of patients exist with lesser degrees of heart failure. Traditional medical therapy for these patients has reached a plateau of benefit and stimulated the development of interventional approaches. Perhaps the best example of such approaches is the creation of an interatrial communication to reduce left atrial pressure, especially in patients with heart failure and preserved ejection fraction. In terms of systolic dysfunction, cardiac contractility modulation by electrical intervention and autonomic interventions involving the vagus and baroreceptors are actively being pursued to alleviate heart failure. Again, these interventions are all notable in patients in whom the major mechanism of failure is left ventricular dysfunction. This is a patient population that has not usually come under consideration by the heart team.

The common theme of the foregoing is the increasing number of interventional procedures for heart failure that are directed primarily to benefit ventricular function rather than structural pathology. This has brought an increasing number of heart failure patients and their physicians to the attention of the heart team. In fact, heart failure specialists who had previously only interacted sporadically with the heart team are progressively becoming regular members of the group. Their presence has clearly enriched the heart team and expanded its role. A recent meeting entitled “THT” for Technology and Heart Failure Therapeutics was recently held entirely on the topic of interventions in heart failure and highlighted in *Structural Heart*. The net result is an increase in the size, scope, vitality, and importance of the heart team, all to the benefit of our patients.

## Funding

The author has no funding to report.

## Disclosure statement

The author reports no conflict of interest.

## References

[1] Wijns W., Kolh P., Danchin N. (2010). Task force on myocardial revascularization of the European Society of Cardiology, the European Association for Cardio-Thoracic Surgery, European Association for Percutaneous Cardiovascular Interventions, guidelines on myocardial revascularization. Eur Heart J.

[2] Alfieri O., De Bonis M. (2010). The role of the edge-to-edge repair in the surgical treatment of mitral regurgitation. J Card Surg.

